# Vasa Vasorum Angiogenesis: Key Player in the Initiation and Progression of Atherosclerosis and Potential Target for the Treatment of Cardiovascular Disease

**DOI:** 10.3389/fimmu.2018.00706

**Published:** 2018-04-17

**Authors:** Daniel G. Sedding, Erin C. Boyle, Jasper A. F. Demandt, Judith C. Sluimer, Jochen Dutzmann, Axel Haverich, Johann Bauersachs

**Affiliations:** ^1^Department of Cardiology and Angiology, Hannover Medical School, Hannover, Germany; ^2^Department of Cardiothoracic, Transplantation, and Vascular Surgery, Hannover Medical School, Hannover, Germany; ^3^Department of Pathology, Cardiovascular Research Institute Maastricht, Maastricht University, Maastricht, Netherlands; ^4^BHF Centre for Cardiovascular Science, Edinburgh University, Edinburgh, United Kingdom

**Keywords:** atherosclerosis, inflammation, vasa vasorum, plaque angiogenesis, unstable plaque

## Abstract

Plaque microvascularization and increased endothelial permeability are key players in the development of atherosclerosis, from the initial stages of plaque formation to the occurrence of acute cardiovascular events. First, endothelial dysfunction and increased permeability facilitate the entry of diverse inflammation-triggering molecules and particles such as low-density lipoproteins into the artery wall from the arterial lumen and vasa vasorum (VV). Recognition of entering particles by resident phagocytes in the vessel wall triggers a maladaptive inflammatory response that initiates the process of local plaque formation. The recruitment and accumulation of inflammatory cells and the subsequent release of several cytokines, especially from resident macrophages, stimulate the expansion of existing VV and the formation of new highly permeable microvessels. This, in turn, exacerbates the deposition of pro-inflammatory particles and results in the recruitment of even more inflammatory cells. The progressive accumulation of leukocytes in the intima, which trigger proliferation of smooth muscle cells in the media, results in vessel wall thickening and hypoxia, which further stimulates neoangiogenesis of VV. Ultimately, this highly inflammatory environment damages the fragile plaque microvasculature leading to intraplaque hemorrhage, plaque instability, and eventually, acute cardiovascular events. This review will focus on the pivotal roles of endothelial permeability, neoangiogenesis, and plaque microvascularization by VV during plaque initiation, progression, and rupture. Special emphasis will be given to the underlying molecular mechanisms and potential therapeutic strategies to selectively target these processes.

## Introduction

As atherosclerotic lesions progress, they can become unstable, and plaque rupture or erosion followed by luminal thrombosis is the primary cause of clinical complications such as myocardial infarction, stroke, and sudden death (1–4). Despite sophisticated interventional and surgical treatment options, the morbidity and mortality from acute cardiovascular events remain unacceptably high. While cholesterol-lowering (5), anti-inflammatory (6), and anti-platelet therapies offer benefits in survival when used in primary or secondary prevention, the benefits of such treatments are still limited and not sufficient in the prevention of acute complications in all treated patients. Specifically, 61% of primary major cardiovascular adverse events are not prevented with current statin treatment regimens in patients as recently shown in the WOSCOPS trial (5). Thus, there is a clear need for novel strategies to both prevent atherosclerotic plaque development as well as to stabilize existing atherosclerotic plaques. Flow-limiting lesions have long been the focus of therapeutic approaches; however, attention has now shifted to the importance of cellular plaque composition rather than the stenotic features alone. Indeed, the cellular features and composition of atherosclerotic plaques have emerged as the most robust predictors of future cardiovascular events (7). Microvessel expansion within the arterial wall and their impact on plaque progression is an area of increasing interest, albeit the precise mechanisms still remain to be determined (8–13). Therefore, the present review will focus on the role of vasa vasorum (VV) neovascularization in atherosclerotic plaque progression and its impact on plaque stability. Furthermore, resulting treatment options focusing on VV neovascularization are discussed.

## Physiological Adaption of VV to the Growth of the Arterial Wall

It was recognized in the early twentieth century that the vessel wall architecture is structurally dynamic and changes with growth and aging (14). At birth, the innermost layer of the vessels is comprised solely of endothelial cells attached to an underlying matrix and surrounded by an internal elastic lamina, while the medial layer has lamellar units consisting of vascular smooth muscle cells (VSMCs), connective tissue, and elastic fibers. The collagen-rich adventitia comprises fibroblasts, perivascular nerves, pericytes, adipocytes, as well as resident leukocyte populations. Due to a pressure gradient, the diffusion of solutes through a permeable medium like the vessel wall is facilitated by the high intra-arterial pressure and is dependent on the permeability of the endothelial layer. With growth, the thickness of the intimal layer increases and the intimal layer gains a higher level of cellular complexity. This process is referred to as diffuse intimal thickening and is now considered a developmental process associated with the growth of the arteries rather than being linked to atherosclerosis itself (15).

While diffusion is responsible for the exchange of nutrients of thin-walled blood vessels, at a critical thickness of more than 0.5 mm, diffusion alone is insufficient (16–18). Hypoxic conditions that arise in the vessel wall of larger blood vessels give rise to VV, defined as arterial microvessels that supply nutrients and oxygen to the adventitia and outer media of the parent vessel.

## Correlation between Arterial Wall Neovascularization and Atherosclerosis Progression

While the thickness of the blood vessel wall is an important parameter governing the neovascularization of VV, other stimuli such as inflammation can trigger neovascularization. For example, even though the murine arterial wall does not exceed the 0.5 mm diffusion limit, VV are seen in atherosclerotic mouse arteries (19–21). In atherosclerotic pigs, vessel wall thickening and plaque development follow the growth of VV in atherosclerotic models (22, 23). The structure of VV is different in non-diseased versus diseased arteries. Early low-resolution X-ray images failed to detect VV in non-diseased human coronary arteries, but in diseased vessels, the presence of a dense microvascular plexus was observed (24). This pattern was also seen in coronary arteries from hypercholesterolemic pigs, where the longitudinal VV externa (defined as first-order VV) originate from the coronary artery as seen in healthy pigs or human arteries. These longitudinal VV further branch to form circumferential arches around the vessel wall, which are defined as second-order VV. Non-diseased porcine coronary arteries display a significantly higher density of first-order VV than the second-order VV. By contrast, the second-order vessel density is twofold greater than the first-order vessel density in hypercholesterolemic pigs (22). Interestingly, the branching patterns of VV reflect the dichotomous tree structure with a hierarchical branching pattern, as seen in the physiological systemic circulation structure. In further findings, Gõssl et al. demonstrated that VV are not connected by a plexus but rather are end arteries (25). Using *ex vivo* micro-CT scans, we could demonstrate that this pathological sprouting pattern can also be observed in VV of small animal models of atherosclerosis, namely, apolipoprotein E^−/−^ (ApoE)/LDLR^−/−^ mice, and can be prevented by an antiangiogenic therapeutic approach (26, 27). Moreover, structural hierarchy in adventitial VV was also later demonstrated *in vivo* in diseased LDLR^−/−^ApoB 100/100 mice by using high-resolution confocal microscopy (28). During VV neoangiogenesis, the branched vessels further branch, occupying the space between two larger vessels. However, in the presence of angiogenesis inhibitors, the newly formed neovessels collapse whereas the larger vessels remain intact. Taken together, data from different animal models demonstrate the presence of some rare, stable, larger VV in healthy vessels. By contrast, under pathological conditions, before and during atherosclerotic plaque progression, neovessels branch out, significantly expand, and exert a disarrayed structure (28).

Thus, microvessels are rarely present in the healthy intima of the vessel wall but are usually observed in pathological conditions such as atherosclerosis. Indeed, a link between atherosclerosis and intraplaque neovascularization was first observed by Koester (29) and Winternitz et al. (30) while the first insights into the mechanism behind the association between atherosclerosis and intraplaque neovascularization was presented by Paterson (31), who was able to identify the rupture of capillaries accompanied by erythrocyte and platelet leakage into the plaque as the cause of plaque progression, rupture, and coronary thrombosis (intraplaque hemorrhage). Further research in the past three decades has largely focused on the role of intraplaque neovascularization in plaque progression and rupture (3, 32–34), confirming the presence of an expansive network of intraplaque neovessels in human stenotic lesions in close proximity to inflammatory infiltration and the necrotic core. Intraplaque hemorrhages are an important trigger for plaque progression, instability, and rupture (3, 35). However, intraplaque neovascularization is also associated with plaque vulnerability and plaque erosion, even in the absence of intraplaque hemorrhage. Moreover, microvessels in the plaque express high levels of cell adhesion molecules (CAM) like intercellular adhesion molecule (ICAM), vascular cell adhesion molecule (VCAM), E-selectin, and cluster of differentiation 40, which facilitate the further recruitment of inflammatory cells into the plaque (36, 37).

One practical example of the importance of VV for vessel integrity and patency can be seen in saphenous veins used for coronary artery bypass graft surgery (38, 39). During the process of saphenous vein harvesting, the connective tissue containing VV is stripped from the vein (38, 40, 41). This often manifests in venospasm (42), which can progress into vein-graft disease and even vein-graft failure, a process analogous to atherosclerosis (40, 43). In addition, venous VV play an important role in vein relaxation, and any damage to the venous VV during saphenous vein harvesting severely impairs flow-induced vasodilation of the graft (43–45).

## VV Expansion and Plaque Angiogenesis

It has been proposed that VV formation occurs as response to the nutritional needs of the artery’s outer medial layer, as the metabolic needs exceed the diffusion levels of oxygen from the luminal blood (17, 18). Under hypoxic conditions, hypoxia-inducible transcription factors (HIF)-1 and HIF-2 induce the transcription of proangiogenic genes like vascular endothelial growth factor (VEGF) (46, 47). Hypoxic conditions in the blood vessel wall also upregulate the expression of important enzymes required for the synthesis of heparan sulfate in microvascular endothelial cells, providing binding sites for fibroblast growth factor-2 (FGF2) (48), which is a known potent stabilizing agent for VV and a promoter of endothelial cell growth (28). In hypercholesterolemic LDLR^−/−^ ApoB100/100 mice, FGF2 is the primary angiogenic growth factor expressed in the adventitial VV, and quantitative polymerase chain reaction measurements have shown an eightfold increase in FGF2 mRNA in hypercholesterolemic mice compared with age- and sex-matched chow-fed mice (21). In hypercholesterolemic mice, FGF2 stimulates the formation of complex VV networks not seen in healthy mice. Tanaka and colleagues delivered FGF2 to the adventitia of ApoE^−/−^ mice demonstrating its role in the expansion of VV and acceleration of plaque progression (49). Other studies investigating the role of placental growth factor (PlGF), a member of the VEGF family of proteins, have shown that the delivery of PlGF into the periadventitial space of the carotid artery significantly increases adventitial neovascularization and macrophage accumulation in hypercholesterolemic rabbits (50). In ApoE^−/−^PlGF^−/−^ mice, the absence of PlGF significantly reduces macrophage accumulation and plaque size (51).

However, and in contrast to the above described “nutritional demand” theory, observations in hypercholesterolemic pigs revealed that VV begin to sprout even before aortic wall thickening and this sprouting is in turn preceded by the infiltration of inflammatory cells into the adventitia (22, 23). Several factors could contribute to the above phenomena, including the secretion of angiogenic growth factors by inflammatory cells infiltrating from the adventitia or by periadventitial fat cells (52, 53). Taken together, it remains to be determined whether hypoxia and nutritional needs, or rather inflammatory stimuli and angiogenic growth factors, induced by the accumulation of pathological particles in the subintimal space, are the initial triggers responsible for VV expansion.

**Figure 1:**
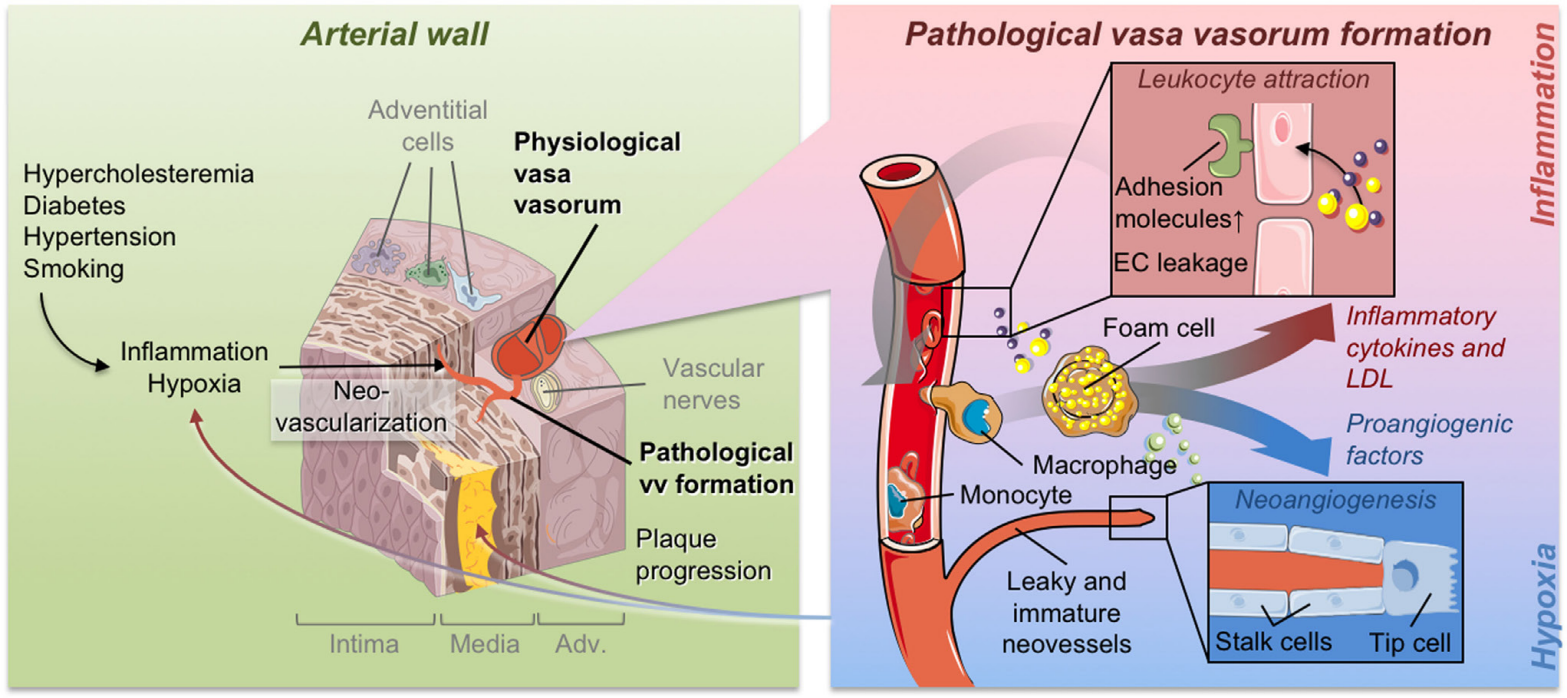
Pathological vasa vasorum formation and its contribution to plaque formation.

While we have gained tremendous insight from the above studies regarding the sequence of pathophysiological events (Figure 1), there are only limited and correlative studies in humans available, suggesting that the infiltration of inflammatory cells into the plaque can be limited by the inhibition of VV angiogenesis and vice versa, resulting in a plaque stabilizing effect (54). Studies in cancer patients revealed a regression of angiogenic blood vessels upon antiangiogenic therapy and, importantly, their reversal to a normal, stable and mature phenotype (55). This normalization of the tumor vasculature is accompanied by a decrease in microvessel density and blood vessel diameter, and at the same time, by an increase in the perivascular cell coverage. As a result, vascular permeability and the interstitial fluid pressure are decreased, resulting in an improved oxygenation of the tumor cells. Hypoxia is a hallmark of solid tumors and drives the production of angiogenic factors. Therefore, by improving tumor oxygenation, normalization of the tumor microvasculature inhibits tumor growth (56). This concept is also valid for VV, where a truncated mutant of plasminogen activator inhibitor-1 (rPAI-1_23_), a potent antiangiogenic protein, has been shown to limit plaque area and plaque volume, and decrease inflammatory cell accumulation and necrotic area, resulting in a reduction in blood vessel stenosis (21, 56). Interestingly, the treatment with rPAI-1_23_ only affects the microvasculature, leaving the larger VV unaffected (28).

## Effects of Cardiovascular Risk Factors on VV Expansion

Studies in experimental animal models on the role of cardiovascular risk factors on VV formation and adventitial remodeling processes are scarce and have yielded varying results (57–59). High cholesterol levels have been associated with an increase in the density of the VV while increased adventitial matrix deposition was observed in hypertensive animals (59). Interestingly, animal models of diabetes mellitus show attenuated growth of VV (60), whereas patients with diabetes mellitus display increased microvessel density, an increased number of inflammatory cells, and more intraplaque hemorrhage (61). Intraplaque hemorrhage, in turn, aggravates atherosclerosis progression due to the increased hemoglobin–haptoglobin complex deposition, which results in oxidative stress-mediated endothelial dysfunction (62). Thus, taken together, there are only limited and partially contradictory data regarding the direct impact of classical cardiovascular risk factors on the development of VV.

By contrast, there is good evidence that chronic inflammation drives angiogenesis, plaque progression, and the occurrence of cardiovascular events. Elevated levels of inflammatory markers are associated with an increased cardiovascular risk (4), and reduced inflammatory levels were shown to yield an equivalent benefit to cardiovascular outcome as the reduction of lipid levels, the classical therapeutic target (63). In humans, a clear correlation between adventitial inflammation, chronic infiltration of CD68^+^ macrophages, and VV expansion was demonstrated (64). Furthermore, perivascular inflammation in autoimmune rheumatoid arthritis, which is associated with an increase in cardiovascular risk, goes along with enhanced VV formation (65).

As a proof of principle, the randomized placebo-controlled multicenter CANTOS trial clinically showed that targeting inflammation potently reduces cardiovascular events. Patients with previous myocardial infarction receiving canakinumab, a monoclonal neutralizing antibody targeting IL-1β, had a significantly lower rate of recurrent cardiovascular events (66). These effects were particularly pronounced in patients with effective reduction of inflammation as evidenced by low C-reactive protein levels during treatment (66). As inflammation is a key trigger of VV expansion, one consequence of dampening inflammation would be to reduce VV neoangiogenesis. Thus, targeting inflammation is a promising strategy to prevent VV growth, plaque progression, and subsequent cardiovascular events.

## Vascular Inflammation as a Trigger for VV Formation—“From Outside In”

Inflammatory mediators are well-known potent triggers of neovascularization in different settings like tumor development or acute ischemic damage [for review see Ref. (67, 68)]. Inflammation and neovascularization tend to feed each other in a vicious cycle in that inflammatory cells in the plaque increase oxygen demand, thereby triggering further neovascularization. Furthermore, as neovascularization progresses, the inherent leakiness of the neovessels, together with the increased expression of adhesion molecules, results in the recruitment of more inflammatory cells into the plaque. Increased expression of angiogenic chemokines like IL-1β have been detected in human atherosclerotic plaques (69) and further increase endothelial cell permeability, allowing the infiltration of leukocytes into the plaque (70). Moreover, continuous inflammatory stimulation causes an irreversible change in endothelial cells to a phenotype consistent with a migratory and proangiogenic state (71).

There was a long-held belief that the infiltration of inflammatory cells, mainly macrophages, occurred through the luminal side of the artery during atherosclerosis progression. By contrast, it has more recently been proposed that vascular inflammation is initiated in the adventitia and progresses toward the media and intima (72). The detection of resident macrophages and T cells in the adventitia further fueled this hypothesis (73). Furthermore, the adventitia is the main site for the acute inflammatory response following vascular injury induced by balloon angioplasty in porcine coronary arteries (74). Adhesion molecules attracting circulating inflammatory cells (VCAM-1 and P-selectin) are expressed most prominently in VV endothelial cells following injury (74). In agreement with these findings, we recently showed that adventitial inflammation is mandatory for the activation of medial smooth muscle cells and subsequent neointima formation (75). Data from a rat model of aortic transplantation between histocompatible strains further supported this hypothesis. In this model, the VV in the adventitia of the aortic allografts triggered a robust angiogenic response in the allograft. Moreover, infiltrating leukocytes were detected in the adventitial VV of the graft, suggesting a role for VV as conduits for the entry of inflammatory cells into the graft (76). Further studies in mice documented the presence of adventitial immune cells already in young wild-type mice, but the number of these adventitial immune cells, especially T-cells, was dramatically increased in hypercholesterolemic ApoE^−/−^ mice (77). Immune cells were further found to organize into tertiary lymphoid structures in the mouse adventitia (73) and are predominantly found in regions next to the external elastic lamina and the atherosclerotic plaque (78).

Studies in humans are consistent with the abovementioned observations in animal experiments. The presence of resident immune cells (T cells, B cells, macrophages, and dendritic cells) in the adventitia was documented in human atherosclerotic arteries (79), and an infiltration of inflammatory cells is observed from the adventitia of the plaque along with the formation of adventitial lymph follicles (80, 81). O’Brien et al. have confirmed the expression of VCAM-1, ICAM-1, and E-selectin, which mediate the recruitment of inflammatory cells, primarily on the intimal and medial VV in human coronary artery segments from patients with atherosclerotic plaques (36, 37). Moreover, leukocytes are present near the adventitial VV even in aortas from healthy children (82). Taken together, there is accumulating evidence that adventitial immune cells play a pivotal role in atherosclerotic disease development and progression and are associated with and probably trigger the growth of VV. However, future research is required to clarify the relevance of this association in human atherosclerosis.

## Increased Permeability of VV Fuels Atherosclerotic Plaque Progression

Endothelial cell junctions are responsible for maintaining vessel wall integrity and preventing the leakage of intravascular components to the extravascular space. Consequently, increased permeability across the endothelial cell layer is an early indicator of vascular dysfunction or the induction of endothelial cell sprouting. As angiogenic processes are initiated, vessel permeability increases, enabling the deposition of serum proteins that form a provisional matrix, triggering and facilitating proangiogenic inflammatory cell adhesion and (trans-) migration. Both local as well as systemic inflammation results in increased permeability of endothelial cell junctions. This process is similar for the early VV expansion phase, where increased endothelial permeability facilitates the infiltration of lipoproteins, inflammatory cells, and red blood cells before and during VV angiogenesis (32). Examination of atherosclerotic coronary arteries in humans showed the lack of mural cells which stabilize endothelial cells and vessel structure and prevent leakage in adventitial VV microvessels invading the medial layer (34). Thus, the sprouting and expanding immature plaque VV are highly fragile and permeable, and thus susceptible to hemorrhage.

Extravasated erythrocytes undergo hemolysis upon exposure to plaque lipids. The released hemoglobin undergoes oxidation and free heme or iron is released, triggering a cycle where hemoglobin interacts with plaque lipids resulting in further oxidation of plaque lipids. Oxidized lipids, in turn, trigger an upregulation of the heme oxygenase-1 in endothelial cells, which catalyzes the formation of active iron, thus further increasing lipid toxicity and endothelial cell damage (83). These events contribute to a thinning of the fibrous cap, making it more prone to rupture (84). This is supported by rabbit data where exogenous erythrocyte injection enhanced plaque progression (85). Moreover, magnetic resonance imaging studies in humans show a direct correlation between intraplaque hemorrhage, plaque growth, and an increase in the volume of the lipid-rich necrotic core (86).

Erythrocytes leaking out into the plaque also act as a source of free cholesterol, increasing the risk of plaque rupture by triggering an influx of macrophages to remove the cell debris (87). Furthermore, the mechanisms regulating the influx and efflux of phagocytic macrophages and clearance of cholesterol, erythrocytic debris, and apoptotic macrophages from the blood vessel walls are largely impaired in advanced atherosclerotic plaques, resulting in the accumulation of free cholesterol from membranes of extravasated erythrocytes in the necrotic core. This, in turn, triggers an inflammatory response and further infiltration of macrophages, resulting in the damage of neighboring cells due to the action of proteases and reactive oxygen species.

To further exploit therapeutic strategies to target pathological VV expansion, reliable preclinical models have to be established which allow an adequate translation of results into the clinical scenario in humans. Unfortunately, most of the available models, especially in small animals, exhibit profound differences in plaque characteristics and VV formation as compared with human arteries and thus suffer from severe limitations regarding their translational relevance. As we will discuss in the following section, only recently have new small animal models been established that more closely resemble the correlation of expanding VV with spontaneous plaque rupture as seen in humans.

## Animal Models to Study VV and Plaque Rupture in Atherosclerosis

Current knowledge on the significance of neovascularization in atherosclerosis is mainly based on studies with human atheroma tissue. A direct association between plaque rupture and intraplaque neovascularization has not been confirmed, as there was a lack of suitable animal models of atherosclerosis. Wild-type rats are particularly resistant to developing atherosclerosis, even on high-fat diets (88) and are therefore not suitable to study plaque neovascularization. As intraplaque microvessels are rare or absent in wild-type pigs, pigs are not often used to study intraplaque neovascularization. However, plaque neovascularization in pigs can be induced with a combination of vascular injury and high cholesterol diets (89). More recently, further pig models have been developed to study intra-plaque neovascularization (90–92). Moreover, a very promisig model of a transgenic Yucatan mini pig overexpressing pro-protein convertase subtilisin/kexin type 9 (PCSK9) showed a more humanized plaque phenotype upon high cholesterol diet. Plaques show intra-plaque and adventitial angiogenesis and micro vessels, although their frequency and microvascular density remains to be determined and quantified (93). More often, intraplaque neovascularization has been investigated in small animal models such as genetically modified mice and rats (94) as well as in rabbits (95, 96).

Atherosclerotic plaques in rabbits are induced by feeding them with a high cholesterol diet in addition to repeated endothelial denudations (97). ApoE^−/−^ or LDLR^−/−^ mice have been extensively used as standard models in atherosclerosis research (98). Moreover, ApoE^−/−^/LDLR^−/−^ double knockout mice kept on a high-fat diet develop plaques rich in lipid content, which until recently, was considered to be the best model of human plaque composition containing foam cells, necrotic cores, and VSMC-rich fibrous caps (99). Moreover, these mice exert an extensive angiogenic activity in the adventitia and develop prominent adventitial and intimal plaque VV (19, 100). Since these mice most closely mimicked atherosclerotic plaques in humans, we and others have used this model extensively during the last years to study VV formation in mice (26, 27, 101). It is commonly supported by cardiovascular pathologists trained in human and experimental pathology (102, 103) that murine plaques most closely resemble human plaque morphology. However, morphology of the recent model of PCSK9-overexpressing mini pig (93) certainly bears strong humanlike features too. Possibly, this is explained by very severe hypercholesterolemia in both PCKS9-mini pigs and apoE/LDLRko models, compared to non-transgenic pigs. However, despite the similarities to human plaque architecture, these models do not develop plaque rupture or thrombosis and are thus unsuitable to investigate correlations of VV formation and plaque instability/rupture.

In a further model utilizing vein grafts in ApoE*3-Leiden mice, the developing atherosclerotic lesions were shown to be even more similar to human plaques, including intraplaque neovascularization together with intimal dissections and intramural thrombus formation (104, 105). Immunostaining studies of histological sections have demonstrated an insufficient pericyte coverage, indicative of leaky and immature microvessels. Importantly, these microvessels were surrounded by extravasated erythrocytes suggesting micro-hemorrhages similar to those found in human plaques (105). Therefore, this model very closely resembles plaque phenotypes in which VV neovascularization is accompanied by intraplaque hemorrhages and plaque ruptures. On the other hand, this model is difficult to establish as it requires a complex vein-graft transplantation surgery and the results are prone to high variability. In a recently established mouse model, a heterozygous mutation (C1039G^+/−^) in the fibrillin 1 gene (*fbn1*) was created, resulting in the fragmentation of the elastic fibers in the medial layer. When back crossed to ApoE^−/−^ mice, the resulting ApoE^−/−^ Fbn1C1039G^+/−^ on a Western-type diet develop spontaneous plaque ruptures (106). Furthermore, these mice spontaneously develop plaques that closely resemble unstable lesions in human plaques, while displaying intraplaque neovascularization and hemorrhages as well as sporadic spontaneous plaque ruptures manifesting into myocardial infarctions (107). Overall, this novel mouse model will facilitate the design of further studies shedding light on the complex interaction of VV formation and plaque progression up to plaque ruptures and the resulting vascular complications. Moreover, this model will be helpful to study novel therapeutic approaches and will allow a better translation efficacy of the gained results into the human/clinical situation.

## Antiangiogenic Strategies in the Prevention and Treatment of Atherosclerosis

**Figure 2:**
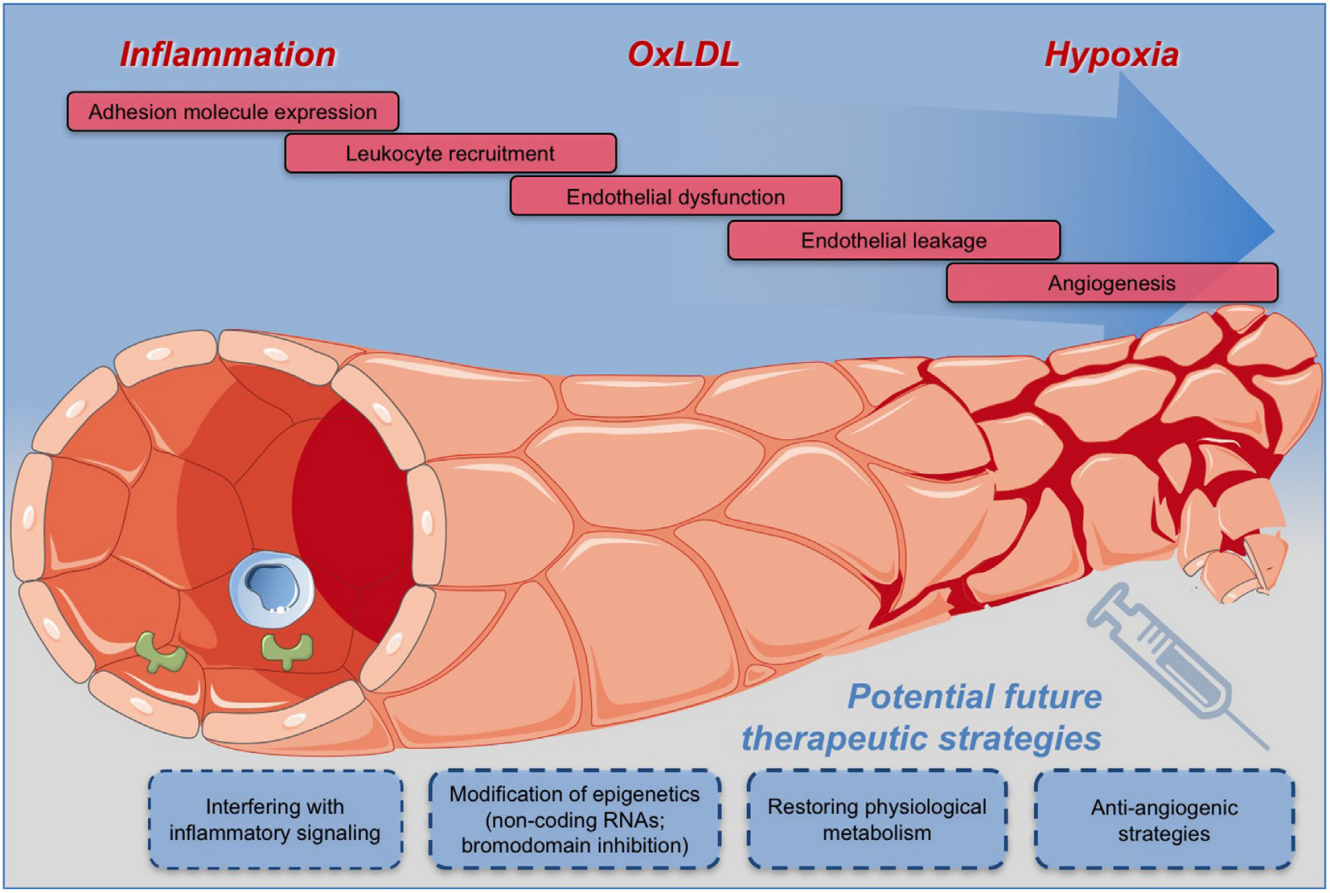
Potential therapeutic strategies for effective and safe targeting of vasa vasorum for the prevention and treatment of atherosclerosis and the related cardiovascular diseases.

As VV and their dysfunction are associated with the initiation and progression of the atherosclerotic process and are later implicated in plaque destabilization, several preventive therapeutic opportunities can be envisioned. One logical consequence would be to target the inflammatory- and angiogenic factors or the endothelial cell response to these factors by targeting cell-specific and cell state-specific signaling events which regulate endothelial cell differentiation, integrity, metabolism, inflammatory- or angiogenic response (Figure 2).

## Inhibiting Vascular Growth Factors

Investigations in animal models have shown that inhibiting vascular growth factors can dampen the expansion of VV. We and others could show that thalidomide (27, 108), endostatin (109), angiostatin (20), angiopoietin-2 (Ang-2) blocking antibodies (110), and rPAI-1_23_ (21, 111) all block VV neovessel formation and slow the progression of atherosclerotic lesions. However, from the cancer field, it is also established that antiangiogenic therapies often have transient effects as there are multiple compensatory mechanisms that take over (112). Therefore, a future strategy could be to combine antiangiogenic factors with anti-inflammatory treatment regimens for the long-term treatment of atherosclerosis.

## Preserving Vascular Integrity

Microvessel quality is determined by its ultrastructural composition: the morphology and integrity of endothelial cells and their junctions, and the extent of pericyte coverage. Defective microvessels are a major source of intraplaque hemorrhage in humans and as already discussed, intraplaque hemorrhage is thought to originate from microvessel hyperpermeability. Restoring microvessel integrity might therefore reduce intraplaque hemorrhage risk and prevent subsequent plaque aggravation. Interesting targets to restore integrity are the main orchestrators of angiogenesis and vascular maturation: VEGF, its receptors, and the angiopoietin family. VEGFs loosen endothelial junctions to allow angiogenic sprouting, which inherently causes vessel leakage in parallel. Several VEGF subtypes, with high affinity for the VEGFR-2 receptor (i.e., VEGF-A and VEGF-F), are able to induce vascular hyperpermeability in a time-dependent and tissue-specific manner (113). VEGF (preferably subtype-specific) inhibition or normalization could be a promising approach to reduce plaque microvessel dysfunction, in addition to its antiangiogenic properties. However, given the extensive physiologically important functions of VEGF, it would be desirable to interfere with downstream players of the VEGF pathway as identified by Laakkonen et al., e.g., SNAI2, RCAN1, MYCN, and NR4A1. Moreover, despite promising effects of VEGF therapy on tumor angiogenesis, serious adverse effects on cardiovascular events (angina pectoris, arterial thrombosis, cerebral- or myocardial ischemia and infarction) have been shown for the VEGF-A inhibitor bevacizumab (114). This is of particular relevance for its potential use as therapy for atherosclerosis, as a history of atherosclerosis greatly enhances the risk of cardiovascular events. The so-called “Janus” face of VEGF is explained by its positive effect on maintenance and regeneration of arterial endothelium, as opposed to its destabilizing effect on microvascular endothelium. Similar concerns for the increased risk of stroke have been described for the intraocular use of the VEGF-A inhibitor ranibizumab (115), suggesting that VEGF inhibition in general should be used with caution.

In addition, Ang-2 increases vascular permeability and decreases pericyte recruitment. Despite the beneficial effects of Ang-2 inhibition in several types of cancer (116, 117), a study examining effects of Ang-2 inhibition in atherosclerosis did not affect murine atherosclerosis and importantly found no impact on adventitial microvessel density (110). Interestingly, angiopoietin-1 (Ang-1), the counterpart of Ang-2, increases the stability of the junctions between the endothelial cells, hence promoting vessel maturity, stability, and reducing leakiness (118). Moreover, it was shown that the balance between Ang-1 and -2 correlates with intraplaque microvessel density in human atherosclerotic plaques, in which the relative abundance of Ang-2 increases microvessel quantity (119).

In addition to endothelial malformations, surrounding pericytes were found to be absent in a majority of microvessels in ruptured plaques (34), clearly linking these cells to plaque destabilization. Unfortunately, little is known about the role of pericytes in endothelial dysfunction and research regarding their relative contribution to plaque development is lacking. From other fields we know that platelet derived growth factor beta (PDGF-B) plays a role in pericyte recruitment at the blood–brain barrier (120, 121). Mice deficient in the PDGF-B retention motif (PDGF-B^ret/ret^) have diminished pericyte coverage leading to permeability of the blood–brain barrier (122). Moreover, the recombinant humanized monoclonal antibody against VEGF-A, bevacuzimab, was shown to reduce vascular leakage by restoring pericyte function through induction of PDGF-B expression *in vivo* in a hindlimb ischemia mouse model (123).

Endothelial barrier integrity is also typically hampered in numerous types of cancer. Firstly, tumor cells constitute an important source of the aforementioned VEGFs. Secondly, pro-inflammatory cytokines like IL-8, often overexpressed by cancerous cells, evoke enhanced endothelial permeability *via* both VEGF-dependent and -independent mechanisms (124, 125). Also, transforming growth factor-β1 (TGF-β1), produced by many cancer type cells in humans (126), is implicated in vascular leakage. *In vitro* data show TGF-β1 inhibits Ang-1 production (127) and stimulates VEGF release (128) in multiple cell lines thereby driving angiogenesis and vascular leakage. Interestingly, in the light of cardiovascular event prevention, the extensively used HMGCoA-reductase inhibitors (“statins”) have shown to increase apoptotic cell death of pericytes both *in vitro* and *in vivo*, possibly counteracting their antiatherogenic features by destabilizing plaques (129). Although pericyte presence in coronary arteries and large blood vessels has been shown (130), their function there remains elusive. However, it is suggested they may play a role in the impairment of adequate microvascular reperfusion after myocardial infarction treatment (131).

In summary, there are several pathways and mechanisms involved in endothelial barrier destabilization, and thus multiple plausible targets to prevent this. VEGF subtypes appear especially interesting as these keep emerging as important players in different pathways. Unfortunately, VEGFs are involved in important physiological processes and therefore perhaps it is not surprising that multiple trials with VEGF inhibiting compounds show also harmful effects (e.g., hypertension, arterial thromboembolic events, and cardiotoxicity). Moreover, as previously addressed, current mouse models are not sufficient for reliably studying the contribution of intraplaque microvessels to plaque aggravation. Hence, advances in animal models to study intraplaque microvessels are needed to gain more insight in this important but underexposed contributor in atherosclerotic plaque development.

## Modulating Endothelial Cell Metabolism to Prevent VV Dysfunction

Endothelial cells have the ability to switch between a mature quiescent state and an angiogenic state. Angiogenesis is an energy-intensive process and requires increased endothelial cell metabolism to support sprouting, migration, and proliferation. Restricting endothelial cell metabolism is a recently recognized strategy that can be used for the inhibition of angiogenesis. Several important recent reviews focus on the strategies that could be used to exploit endothelial metabolism for the development of antiangiogenesis therapy (132–135). As angiogenic endothelial cells rely heavily on glycolysis for ATP generation (136), inhibitors of the key glycolysis enzyme 6-phosphofructo-2-kinase/fructose-2,6-biphosphatase 3 (PFKFB3) have been shown to maintain endothelial cells in a quiescent state, reducing injury- and inflammation-induced pathological angiogenesis *in vivo* (135, 137).

Upregulation of CAM has been observed in VV endothelial cells, facilitating the recruitment of circulating leukocytes. In tumor models, inhibition of PFKFB3 impairs nuclear factor kappa B (NF-κB) transcriptional activity in endothelial cells by targeting the phosphorylation of p65 and IκBα, which in turn decreases CAM expression (138). As VV play an important role in the recruitment of inflammatory cells into atherosclerotic plaques, specific reduction of CAMs by a PFKFB3 inhibitor could impact plaque initiation and progression.

We suspect that modulating endothelial cell metabolism could be used to develop strategies to stabilize VV neovessels and therefore might be of help in controlling the initiation and progression of atherosclerosis. Pericytes near the parental endothelial layer are known to surround the VV microvessel endothelium, stabilizing it, and thus establishing a mature, non-leaky vessel phenotype (139). Studies in cancer models have shown that PFKFB3 inhibitors can decrease pericyte glycolysis and impair their migration and proliferation. PFKFB3 inhibitors also promote cell quiescence and tighter cell–cell junctions (138), resulting in a tighter pericyte layer covering the endothelial cell layer and leading to the maturation and normalization of the tumor vasculature. Thus, targeting pericyte cell metabolism could be advantageous in stabilizing VV structure (13, 140, 141).

Experimentally used glycolysis inhibitors result in transient and incomplete inhibition of glycolysis. To maintain cell homeostasis, glycolysis flux is required to avoid detrimental systemic side effects (137, 142). And in fact, it can be assumed, that blocking VV neoangiogenesis could create an environment that is highly hypoxic and could worsen cell necrosis and promote plaque development. Therefore, incomplete or partial inhibition of glycolysis might very likely be advantageous in this setting.

## Epigenetic Modification to Render Endothelial Cells Less Sensitive to Inflammatory Stimuli

Epigenetic regulation of gene expression *via* DNA methylation and histone posttranslational modifications can modulate gene expression by affecting the binding of specific transcription factors. Recent research has revealed the role of several epigenetic modifications in the pathology of atherosclerosis. These modifications are specific to particular cell types and affect specific stages of the disease (143, 144). The following section elaborates the involvement of two specific inflammatory triggers that result in epigenetic modifications in atherosclerosis and the mechanisms that could be specifically targeted to reduce the sensitivity of the VV endothelial cells to inflammatory triggers. Exposure of endothelial cells to oxidized low-density lipoproteins (oxLDL) upregulates expression of DNA methyltransferase (DNMT) 1. DNMT1 then methylates the promoter encoding the anti-inflammatory transcription factor Krüppel-like factor 2 (KLF2), resulting in its repression. Inhibition of DNMT1 by 5-aza-2′-deoxycytidine prevents methylation of the KLF2 promoter (145), and therefore this strategy could suppress the response of the endothelium to oxLDL by blocking the inflammatory and angiogenic signaling mechanisms.

Brown et al. recently uncovered the signaling mechanisms involved in inflammatory cytokine-mediated epigenetic changes that occur in endothelial cells. These epigenetic changes further drive the inflammatory processes (143). When endothelial cells are exposed to tumor necrosis factor α (TNFα), the transcription factor NF-κB locates to enhancers and promoters genome-wide, where it recruits bromodomain-containing protein 4 (BRD4). Through the recruitment of BRD4, TNFα rapidly induces new super enhancers (inflammatory super enhancers) that drive NF-κB-mediated pro-inflammatory gene expression. Inhibiting BRD4 in endothelial cells decreases the expression of pro-inflammatory cytokines and CAM and attenuates leukocyte extravasation and plaque burden in a mouse model of atherosclerosis. Similarly, inhibition of bromodomain and extra terminal domain (BET) proteins, as occurring in BRD4, by the use of specific inhibitors like, i.e., I-BET and JQ1, have also shown positive results by dampening endothelial inflammation (146).

Epigenetic changes also regulate endothelial cell responses to hypoxia, as has been shown in cancer biology. BET inhibitors impair endothelial cell response to hypoxia thereby reducing hypoxia-induced angiogenesis (147). Investigations adapting these mechanisms to atherosclerosis research would elucidate the role of BET inhibitors in modulating the response of VV to hypoxic conditions.

Investigation of the specific responses that occur in VV under hypoxic and inflammatory conditions would help identify the best target molecules for therapeutic modalities. Similarly, specific targeting of epigenetic regulators of the neovasculature could provide a method for site-specific targeting to maintain the VV endothelial cells in a quiescent and mature state.

## Therapeutic Possibilities of MicroRNAs (miRNAs) to Tackle VV Dysfunction

MicroRNAs are small non-coding RNAs that can target multiple genes and inhibit gene expression. miRNA dysfunction is associated with atherosclerosis (148), and multiple different miRNAs, as well as target genes, can affect the progression of atherosclerosis. The therapeutic use of miRNAs is complicated by the fact that each miRNA can target mRNA of several different, completely unrelated genes and these effects may further be influenced by the cell type, its activation and/or differentiation state, and its microenvironment (149). miR-126 is thought to have atheroprotective effects as it reduces the inflammatory response by decreasing the expression of leukocyte adhesion molecules and inhibits angiogenesis in mature endothelial cells (150). However, under hypoxic conditions, or in the case of a injured vessel wall, miR-126 stimulates the formation of neovessels, thereby assuming a proangiogenic function (151–153). Therefore, given the hypoxic conditions in the VV, upregulation of miR-126 may have adverse effects assuming a proatherogenic response, resulting in a localized proliferation of unstable neovessels. Other miRNAs identified with potential therapeutic uses include miR-221 and miR-222 (154). While miR-221/222 support endothelial quiescence, they also downregulate endothelial nitric oxide synthase (155), causing endothelial dysfunction. miRNA-221/222 also simulate VSMC proliferation and accelerate neointima formation (156), which is a contributing factor for plaque progression.

Inhibition of endothelial cell-specific miRNAs like miR-92a augments angiogenesis during cardiac regeneration (157) and has a favorable effect on re-endothelialization and neointima formation after vascular injury (158). Special features of miRNAs such as cell-specific expression and cell- and activation state dependent regulation make them attractive targets for precise therapeutic approaches. While miRNA-targeting strategies hold a valid therapeutic potential, their feasibility, safety, and effectiveness in the prevention and treatment of atherosclerosis will have to be determined.

## Conclusion and Future Perspectives

Substantial scientific evidence documents a clear association between the expansion of VV and plaque neovascularization with atherosclerotic plaque growth and progression toward an inflammatory and unstable plaque phenotype leading to plaque rupture and related clinical events. In physiological conditions, VV enable the access of oxygen and nutrients to the vessel wall. However, when expanding due to pathological stimuli, VV set the milieu for plaque growth and function as carriers of cholesterol, inflammatory cells, erythrocytes, provisional extracellular matrix, or other atherogenic molecules into the growing plaque (Figure 1). Conversely, prevention of new VV growth or stabilization of existing physiological VV was documented to be followed by a reduction in plaque growth and increased plaque stability. Despite these clear associations, there is a gap in the knowledge regarding the precise mechanisms regulating pathological VV expansion.

Consequently, therapeutic approaches specifically targeting the expanding microvessels in developing plaques will have to be established and evaluated. In particular, advances in the understanding of the metabolic and epigenetic players involved in the regulation of disease-specific functions of endothelial cells will enable the development of new treatment modalities for an effective and safe targeting of VV and thus for the prevention and treatment of atherosclerosis and the related cardiovascular diseases (Figure 2).

## Author Contributions

DS, EB, JAFD, and JS performed literature search and analysis and wrote the content of the manuscript. AH and JB contributed by editing and proofreading. JD designed the figures and edited the manuscript.

## Conflict of Interest Statement

The authors declare that the research was conducted in the absence of any commercial or financial relationships that could be construed as a potential conflict of interest.

## Abbreviations

ApoE, apolipoprotein E; BET, bromodomain and extra terminal domain; BRD4, bromodomain-containing protein 4; CD40, cluster of differentiation 40; DIT, diffuse intimal thickening; Fbn, fibrillin; FGF2, fibroblast growth factor-2; HIF, hypoxia-inducible transcription factors; ICAM, intercellular adhesion molecule; LDL, low-density lipoprotein; LDLR, low-density lipoprotein receptor; PFKFB3, 6-phosphofructo-2-kinase/fructose-2,6-biphosphatase 3; PlGF, placental growth factor; qPCR, quantitative polymerase chain reaction; ROS, reactive oxygen species; rPAI-1, recombinant plasminogen activator inhibitor-1; TGF, transforming growth factor; TNFα, tumor necrosis factor α; VCAM, vascular cell adhesion molecule; VEGF, vascular endothelial growth factor; VSMCs, vascular smooth muscle cells; VV, vasa vasorum.
